# Preparation of Borders of Cavities for Filling

**Published:** 1898-10

**Authors:** R. B. Howell

**Affiliations:** Ann Arbor, Mich.


### Preparation of Borders of Cavities for Filling.

BY R. B. HOWELL, D.D.S., ANN ARBOR, MICH.

This subject is, without doubt, one of the most important subjects in operative dentistry; indeed, it may be said that on the proper management of same rests the ultimate success of an operation in filling.

The essential qualities of a good filling are: 1st. Perfect adaptation to borders of cavity so as to exclude moisture and all corrosive agents. 2d. A good, smooth finish to edges of filling which will lessen the chance for lodgment of foreign substances.

To bring about these all-momentous attributes one must have a perfect knowledge of the structure upon which he works, and not bow to the old maxim, "Experience is the best teacher," but study carefully and thoroughly all things which will tend to broaden his knowledge of this branch of his profession. In other words, be conscientious, do not make the patient pay for your experience.

What are the requirements necessary for this?

Since the borders of cavities are necessarily made upon the enamel, we must be thoroughly familiar with all the properties of this constituent of the tooth.

It is not the object of this paper to enter into a discussion on structure of enamel, as we are all conversant with same, suffice it to say the enamel rods usually run perpendicularly from the dentine but are quite irregular in malformed teeth; in fact, it is often very difficult to determine satisfactorily the direction of enamel rods, as they are not absolutely the same on similar parts of similar teeth.

Upon the direction of these rods is placed our foundation for a perfect border, as it determines the line of cleavage.

In breaking down enamel you have no doubt, noticed that there are certain lines which it follows, these are the lines of cleavage.

In order to produce perfect borders to completed fillings we must so form the enamel borders of the cavity that they shall present the strongest edge possible against which to condense the filling material. If the enamel was equally strong in all directions a square edge would be the one, but on account of cleavage this form is rendered unsafe as the dentinal ends of the rods will be left without support, and since the cement substance between enamel rods is so much more soluble than the rods the latter will be disintegrated and will drop off, thus leaving an opening for contamination.

This difference in solubility of rods and cement substance also has an important bearing on the management of borders of cavities. If acid be added to a portion of enamel the rods will be separated from each other; the same thing occurs in caries of the teeth when decay enters through the enamel into the dentine it will dissolve the enamel away from underneath and we have a kind of secondary decay from within out, it also spreads laterally underneath enamel, so in breaking down the walls of a cavity we should be very careful to follow the decay until the border is found to rest on solid dentine. After this trim the edges parallel with the enamel rods through its entire thickness and make as smooth as possible by carefully shaving with a sharp excavator.

It is best to cut lengthwise with the enamel rods, for by cutting from within out or without in, the rods will be splintered up and broken off in little chunks, thus leaving irregular openings to which it will be impossible to adapt a filling, and by this carelessness invite recurrence of caries.

The next procedure is to bevel the extreme edges with a sharp excavator so as to give a good, strong edge to the filling. The edge of border should be beveled and not rounded, the latter is brought about by the use of the disc, which has become quite universal in the hands of operators; it is next to impossible to make a bevel edge with a disc. The rounded border leaves a little tongue of gold, or I might say feather edge, extending over the border, which is very readily broken off, much to the detriment of a good filling.

Another important factor in the preparation of borders of cavities is the old and somewhat trite saying, "Extension for prevention."

There are certain lines in the teeth where the enamel is singularly weak, these are the developmental grooves in teeth. In order to understand these lines it is necessary to review the development of teeth.

As you all know there are four points of beginning in the formation of incisors, cuspids and bicuspids and three to five in the molars, according to the number of cusps. You are also familiar with the malformation due to the improper union of these lobes or centers. Examination and experimentation has shown that in these lines the enamel has weaker consistency than in other portions of the tooth.

If a patient entered your office and desired to have a filling made in a molar tooth, which cantained several small cavities in the crown, you would not hesitate to run them together into one large filling in order to prevent further decay, yet in approximal cavities of bicuspids and molars, the above theory is not carried out.

Whenever a border would leave a small portion of enamel between it and any of the developmental grooves, cut to the groove and form the border of cavity along same or just beyond it. The most frequent errors of this kind are made at the disto-buccal groove on the lower first molar, the triangular grooves of bicuspids, disto-lingual of upper molars and the grooves on edges of incisors and cuspids.

These seem, to the writer, to be the important points in the preparation of borders of cavities for filling.

In summing up, will say: (1) Cut the enamel to such a point that the surface of the filling may be so formed that the enamel border will be self-cleansing or be protected by the gum margin, and never, under any circumstances, allow enamel border to make an approximate contact. (2) Do not form the border in such a position as to leave a small portion of the enamel between it and one of the developmental grooves. (3) Do not so form the border that it will present any short ends of enamel rods on its outer edge, and do not make bevel of same so great that the filling will not have a good edge strength.

